# Engineering a Smart Agent for Enhanced Immunotherapy Effect by Simultaneously Blocking PD‐L1 and CTLA‐4

**DOI:** 10.1002/advs.202102500

**Published:** 2021-09-02

**Authors:** Chunjuan Jiang, Le Zhang, Xiaoping Xu, Ming Qi, Jianping Zhang, Simin He, Qiwei Tian, Shaoli Song

**Affiliations:** ^1^ Department of Nuclear Medicine Fudan University Shanghai Cancer Center Shanghai 200032 China; ^2^ Center for Biomedical Imaging Fudan University Shanghai 200032 China; ^3^ Shanghai Engineering Research Center of Molecular Imaging Probes Shanghai 200032 China; ^4^ Department of Research and Development Shanghai Proton and Heavy Ion Center Shanghai 201321 China; ^5^ Shanghai Key Laboratory of Molecular Imaging Shanghai University of Medicine and Health Sciences Shanghai 201318 China

**Keywords:** ^19^F MRI, granzyme B probes, immunotherapies, positron emission tomography/computed tomography imaging, ZIF‐8

## Abstract

Combinations of immune checkpoint therapies show encouraging results in the treatment of many human cancers. However, the higher costs and greater side effects of such combinations compared with single‐agent immunotherapies limit their further applications. In this work, a novel smart agent, KN046@^19^F‐ZIF‐8, is developed to overcome these limitations. KN046 is a novel recombinant humanized PD‐L1/CTLA‐4 bispecific single‐domain antibody‐Fc fusion protein, which can bind to both PD‐L1 and CTLA‐4 effectively. ZIF‐8 is a smart delivery system, which can safely and effectively deliver KN406 to a tumor. In vitro and in vivo results demonstrate that the smart agent KN046@^19^F‐ZIF‐8 not only improves the immune response rate of the antibody drug in treatment of tumors but also reduces its toxic side effects, thereby achieving excellent antitumor efficacy. This study provides an engineering strategy for clinical applications of a more effective immunotherapy.

## Graphical abstract

A novel smart agent is engineered by coating a tumor microenvironment responsive ZIF‐8 on the novel recombinant humanized PD‐L1/CTLA‐4 bispecific single‐domain antibody‐Fc fusion protein to overcome the higher costs and greater side effects of single‐agent immunotherapies.

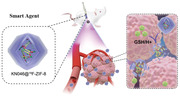

## 1 Introduction

Immune checkpoint therapy to improve T cell activity has shown encouraging progress in the treatment of many human cancers,^[^ ^1^, ^2^, ^3^ ^]^ especially malignant and chemotherapy‐resistant cancers.^[^ ^4^, ^5^ ^]^ Among the immune checkpoint inhibitors used in this type of therapy, CTLA‐4 and PD‐1 antibodies have emerged as the most effective agents for activation of antitumor immune responses and have been approved by the Food and Drug Administration to treat many cancers.^[^ ^3^, ^6^ ^]^ However, it has proved difficult to achieve an objective response rate of more than 20% with single‐agent immunotherapies.^[^ ^7^ ^]^ Consequently, a combination of CTLA‐4 and PD‐1 antibodies, used to simultaneously block PD‐L1 and CTLA‐4, was proposed to improve response rates. In 2015, a combination of Opdivo (a PD‐1 antibody) and Yervoy (a CTLA‐4 antibody) was approved to treat metastatic melanoma. The clinical results were encouraging, with 72% of patients in the combination treatment group still alive after 5 years.^[^ ^8^ ^]^ Unfortunately, such combinations of drugs are not only more expensive but are also associated with more side effects than single‐agent immunotherapies. Therefore, it remains a great challenge to develop low‐value and high‐efficiency immunotherapy drugs and treatments.

Simplifying immune antibodies to retain only their effective sites is the main strategy to reduce their side effects and costs. Based on this approach, a novel recombinant humanized PD‐L1/CTLA‐4 bispecific single‐domain antibody‐Fc fusion protein (KN046) was designed to bind to both PD‐L1 and CTLA‐4 and effectively enhance the killing of tumor cells. To the best of our knowledge, few studies have explored the treatment effectiveness of such PD‐L1/CTLA‐4 fusion proteins. In addition, immune antibodies after intravenous administration cannot effectively accumulate in the tumor caused by off‐target effects,^[^ ^9^, ^10^ ^]^ so the bioavailability tends to be low. Therefore, engineering is required in order to safely and effectively deliver KN406 to tumors and exploit its therapeutic effects.

Smart nano‐delivery agents with highly biocompatible and biodegradable properties can not only protect the active ingredients of an immunotherapy from damage but can also accurately deliver the antibody to the tumor area. ZIF‐8, which is a metal–organic framework formed by zinc (Zn^2+^) and imidazole,^[^ ^11^, ^12^ ^]^ is a widely used smart nano‐delivery agent, as it can be rapidly degraded by weakly acidic and highly expressed glutathione (GSH) in tumors.^[^ ^13^, ^14^, ^15^ ^]^ For example, An et al.^[^ ^16^ ^]^ reported an engineered smart agent based on ZIF‐8‐coated gold nanospheres. In normal tissues, the ZIF‐8 coating is very stable and prevents the aggregation of the gold nanospheres; however, the nanospheres are released in the tumor owing to destruction of ZIF‐8. Therefore, coating KN046 with ZIF‐8 could reduce adverse reactions in normal tissues, improve its enrichment in tumors, and prevent its clearance by the immune system.

**Figure 1:**
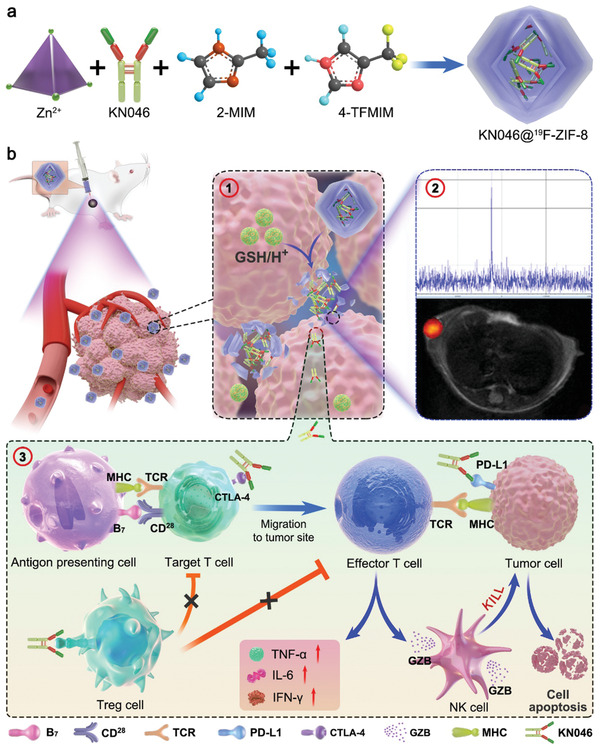
Schematic illustration of the formation of KN046@^19^F‐ZIF‐8 nanoplatform (a) and the mechanism of dual‐blockade of an immune checkpoint to enhance melanoma immunotherapy (b). ① Structure changes of ^19^F‐ZIF‐8in response to the tumor microenvironment invitro. ② ^19^F NMR signals in response to GSH/H+. ③ In vivo immune responsesof dual‐targeting inhibitor of immune checkpoints.

As a proof of concept, a smart nano‐agent, KN046@ZIF‐8, was designed to explore the enhanced immunotherapy effect of simultaneously blocking PD‐L1 and CTLA‐4 (**Figure** **1**a). In order to monitor the release of antibodies in real time, fluorine was doped in ZIF‐8 to form a smart ^19^F‐MRI probe, which is turned on by the F‐MRI signal when ZIF‐8 is destroyed. The KN406 antibody is protected by ZIF‐8 in normal tissue but released in tumors to block the binding of PD‐L1 to PD‐1 and CTLA‐4 to CD80/CD86, thereby effectively enhancing the killing of tumor cells (Figure 1b). In addition, for non‐invasive, real‐time evaluation of the effects of KN046@^19^F‐ZIF‐8, ^68^Ga‐NOTA‐GZP (Figure S1, Supporting Information), and ^18^F‐FDG positron emission tomography (PET)/computed tomography (CT) were used for real‐time monitoring of granzyme B, which reflects the immune activation state in the tumor.

## 2 Results and Discussion

### 2.1 Synthesis and Characterization of KN046@^19^F‐ZIF‐8

**Figure 2:**
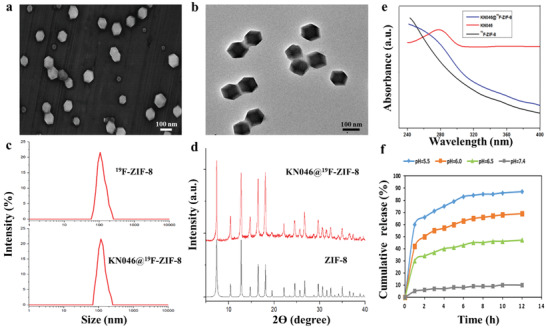
Characterization of KN046@^19^F‐ZIF‐8. a) SEM image of KN046@^19^F‐ZIF‐8. b) TEM image of KN046@^19^F‐ZIF‐8. c) Size distribution of ^19^F‐ZIF‐8 and KN046@^19^F‐ZIF‐8. d) XRD patterns of KN046@^19^F‐ZIF‐8 and ZIF‐8. e) UV–vis absorption spectra of as‐prepared ^19^F‐ZIF‐8, KN046, and KN046@^19^F‐ZIF‐8. f) KN046 release profiles from KN046@^19^F‐ZIF‐8 in PBS at pH values of 5.0, 6.0, 6.5, and 7.4.

Prior to formulation, surface modification of KN046 was performed using polyvinylpyrrolidone (PVP). Subsequently, the modified KN046 was encapsulated in the fluorine‐containing ZIF‐8 nanoshell using an improved ZIF‐8 synthesis technique (Figure 1a). The whole process was carried out in ultrapure water, with a short reaction time, mild reaction conditions, and high yield. Scanning electron microscopy (SEM) (**Figure** **2**a) and transmission electron microscopy (TEM) revealed that the KN046‐loaded ^19^F‐ZIF‐8 was hexagonal, regular in shape, and uniform in size (Figure 2b). The TEM images and crystal structure analysis of X‐ray diffraction (XRD) also showed that the structure was destroyed in a mildly acidic environment (Figure S2, Supporting Information). The particle size of ^19^F‐ZIF‐8 as measured by dynamic light scattering (DLS) was 60–250 nm, whereas that of the KN046‐loaded ^19^F‐ZIF‐8 was increased to 70–260 nm with an average size of 112.4 ± 4.7 nm (Figure 2c). This size makes it suitable for medical applications. Meanwhile, KN046@^19^F‐ZIF‐8 exhibited good dispersion stability in water (Figure S3, Supporting Information). The zeta potential of ZIF‐8 decreased from 13 to −1 after fluorine was replaced (Figure S4, Supporting Information). XRD showed that the spectrum of the KN046@^19^F‐ZIF‐8 nanostructure contained diffraction peaks corresponding to ZIF‐8 (Figure 2d), showing that formation of ^19^F‐ZIF‐8 did not destroy the structure of ZIF‐8. The KN046@^19^F‐ZIF‐8 nanostructure showed an absorption peak at 280 nm according to UV spectrophotometry (Figure 2e), consistent with the characteristic peak of the pure KN046 antibody at 280 nm, indicating that KN046 was successfully embedded in the ^19^F‐ZIF‐8 nanoshell. The encapsulation efficiency (EE) of KN046 in the KN046@^19^F‐ZIF‐8 nanoparticle was 40.8% ± 4.31% and the loading efficiency was 3.2% ± 0.87%, as determined by high‐performance liquid chromatography (HPLC). It is well known that the structure of ZIF‐8 will decompose in an acidic environment or in the presence of GSH at high concentrations. The drug release curve showed that KN046 remained basically stable in the KN046@^19^F‐ZIF‐8 nanoparticle at pH = 7.4. However, in a mildly acidic environment, KN046 release occurred in two stages: rapid release in the early stage, followed by sustained release over time from the nanoshell. The cumulative release rate at day 12 was 85% ± 3.54% in buffer solution at pH = 5.5 (Figure 2f). These results indicated that KN046@^19^F‐ZIF‐8 could be responsive to the tumor microenvironment and enable controlled release of KN046.

### 2.2 Changes in ^19^F NMR Signals in Response to GSH/H^+^

**Figure 3:**
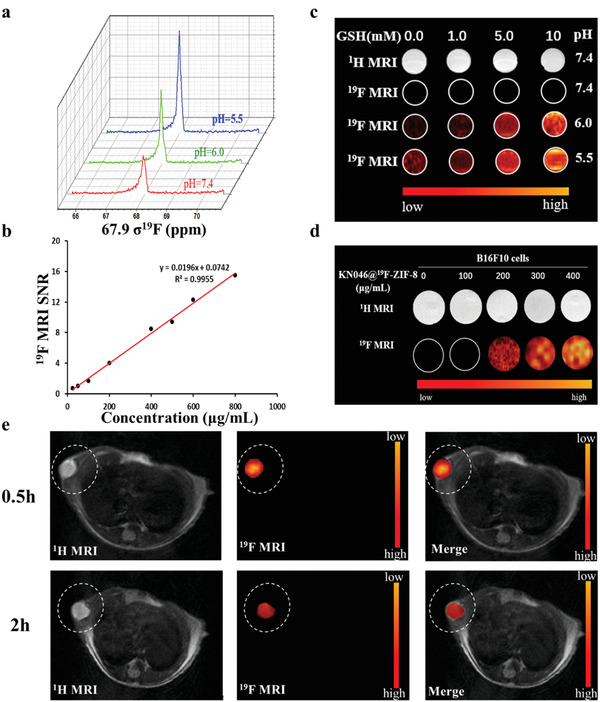
a) Normalized ^19^F‐MR spectra as a function of pH value for KN046@^19^F‐ZIF‐8_3mg_. b) Calibration plot of the ^19^F‐MR SNR (signal‐to‐noise ratio) versus KN046@^19^F‐ZIF‐8 concentration in the region of 25–800 µg mL^−1^. *C*_GSH_ = 10 mm at pH = 6.0. (*C*_GSH_ represents the concentration of GSH.) c) ^19^F NMR signal intensity of KN046@^19^F‐ZIF‐8_3mg_ solution under different pH values and GSH concentrations (0, 1.0, 5.0, and 10.0 mm). d) ^1^H MRI and corresponding ^19^F MRI images of B16F10 cell lysates with different concentrations of KN046@^19^F‐ZIF‐8 at 6 h. e) In vivo ^1^H/^19^F MRI and merged images of a B16F10 tumor‐bearing mouse after intratumoral injection of KN046@^19^F‐ZIF‐8 solution (20 mg mL^−1^, 150 µL) at 0.5 and 2 h. ^1^H MRI was conducted using a T2‐weighted imaging method.

The molecular imaging system and the controlled release of immune antibodies can be combined to accurately diagnose tumors and monitor the release of antibodies in real time.^[^ ^17^, ^18^ ^] 19^F MR imaging is a powerful supplement to conventional ^1^H MRI due to high sensitivity and negligible background signal.^[^ ^19^, ^20^, ^21^ ^]^ Three pH values (7.4, 6.0, and 5.5) and GSH concentrations between 1.0 and 10 mm were used for studies based on the physiological and tumor microenvironmental characteristics. The ^19^F MRI response of KN046@^19^F‐ZIF‐8 was evaluated at different pH values and GSH concentrations. **Figure** **3**a shows ^19^F MR spectra of the nanoprobe solution under different pH conditions. A single sharp peak centered at −67.9 ppm was observed. The intensity of this was very low at pH 7.4. However, ^19^F MRI signal intensity of the nanoprobe solution increased as the pH decreased. When the pH was lowered to 6.0 or 5.5, the coordination bond between Zn^2+^ and methylimidazole/trifluoromethylimidazole dissociated, thereby releasing ^19^F from restricted motion. At this point, the intensity of the peak at −67.9 ppm was significantly enhanced. This demonstrates the pH responsiveness of the prepared metal–organic framework‐based ^19^F nanoprobe. Moreover, in an acidic environment (pH 6.0 or 5.5), the ^19^F MRI signal intensity increased as the GSH concentration increased (Figure 3b). In a physiological environment with a pH above 7, the ^19^F MR signal was negligible even at the highest GSH concentration (10 mm). However, in an accurately simulated tumor microenvironment in vitro (weakly acidic with high GSH concentration),^[^ ^22^, ^23^, ^24^ ^]^ the nanoprobe solution provided high‐contrast ^19^F MR images (Figure 3c). These results indicate that the response of the probe to GSH/H^+^ conditions is highly specific to the tumor microenvironment.

### 2.3 In Vitro Characteristics

The cytotoxicity of the nanoprobe was evaluated by CCK‐8 assay. B16F10 cells were incubated with different concentrations of the nanoprobe for 48 h (Figure S5, Supporting Information). The viability of B16F10 cells remained above 80% after 48 h, even when the concentration of the nanoprobe was 800 µg mL^−1^, demonstrating the high biocompatibility of the KN046@^19^F‐ZIF‐8 nanoprobe. Next, fluorescent orange rhodamine B was loaded into ^19^F‐ZIF‐8 instead of KN046. High‐intensity orange fluorescence in the tumor cytoplasm was observed by confocal laser scanning microscope (CLSM) after incubation with B16F10 cells for 2 h. The intensity of orange fluorescence decreased slightly at 4 h (Figure S6, Supporting Information), demonstrating the effective phagocytosis of tumor cells by the nanoparticles. Furthermore, the ^19^F MR response of the KN046@^19^F‐ZIF‐8 nanoprobe in B16F10 cells was evaluated. After incubation with different concentrations (0, 100, 200, 300, and 400 µg mL^−1^) of the nanoprobe for 4 h, the B16F10 cells were lysed. The imaging performance of the lysate was analyzed by ^19^F NMR. As shown in Figure 3d, the ^19^F MR signal became brighter as the concentration of the nanoprobe increased.

### 2.4 In Vivo ^19^F MR Response

The nanoprobe solution was injected into a transplanted subcutaneous melanoma model on the right sides of mice by intratumoral injection. High‐intensity ^19^F MR imaging was obtained 0.5 h after injection. The ^19^F MRI signal intensity gradually decreased 2 h after injection (Figure 3e). Traditional ^1^H MRI clearly showed anatomical structures and tumor contours, whereas ^19^F MRI only showed high‐contrast images at the tumor site,^[^ ^25^, ^26^ ^]^ with negligible fluorine signal in normal tissues. The above findings indicate that the ZIF‐8‐based ^19^F‐MRI nanoprobe responds quickly in the tumor microenvironment, allowing adequate migration of ^19^F to ensure sufficient fluorine content and activate the ^19^F MR signal in a shorter time. Therefore, a ^19^F‐ZIF‐8 MRI probe that responds to the tumor microenvironment could effectively avoid signal interference from the normal body. It could distinguish between tumors and normal tissues more sensitively and intuitively to allow tumor‐specific imaging and improve the signal‐to‐noise ratio, thereby providing better imaging support for tumor diagnosis. This provides a basis for clinical transformation of the ^19^F‐MRI probe.

### 2.5 In Vivo Efficacy and Immune Response

**Figure 4:**
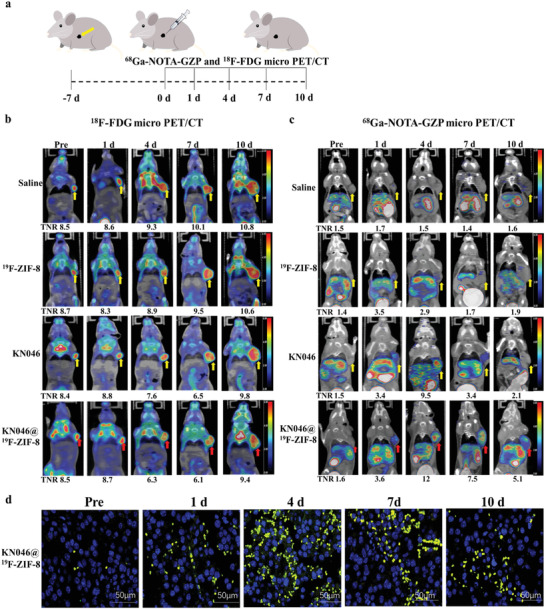
a) Schematic diagram of the therapeutic model and micro PET/CT imaging in BALB/c mice bearing subcutaneous B16F10 melanoma tumors. Representative b) ^18^F‐FDG and c) ^68^Ga‐NOTA‐GZP micro PET/CT images at different times after different treatments. d) Granzyme B immunofluorescence images of the tumors at different time points after KN046@^19^F‐ZIF‐8 treatment (pictured at 200×) (*n* = 5).

Before the formal experiments, PD‐L1 expression in B16F10 melanoma tumor tissue was confirmed by the observation of strongly positive immunohistochemical (IHC) staining (Figure S7, Supporting Information). A subcutaneous implantation of B16F10 melanoma was generated in BALB/c mice to evaluate the efficacy of the nanoprobe in the treatment of melanoma and examine the immune response induced by treatment. The mice with subcutaneous B16F10 tumors (primary tumors only) were divided into four groups to receive normal saline, ^19^F‐ZIF‐8, KN046, or KN046@^19^F‐ZIF‐8 (**Figure** **4**a).

#### 2.5.1 ^18^F‐FDG and ^68^Ga‐NOTA‐GZP PET/CT for Assessing Expression of Granzyme B in Tumors after Immunotherapy

Diameter measurements by structural imaging techniques such as CT or MRI are not appropriate to assess the efficacy of immunotherapy. ^18^F‐FDG PET/CT is a recognized method for diagnosing neoplasms and monitoring efficacy.^[^ ^27^, ^28^ ^]^ However, ^18^F‐FDG PET/CT only reflects the glucose metabolism of tumors,^[^ ^29^, ^30^ ^]^ which will result in pseudoprogression and hyperprogression due to the inflammatory infiltration induced by immunotherapy. Granzyme B is a serine protease released from cytoplasmic granules in cytotoxic T cells (CTLs) and natural killer cells. It initiates the caspase cascade to induce DNA degradation in tumor cells.^[^ ^31^, ^32^ ^]^ As a powerful killing factor in cellular immunity, granzyme B reflects the immune activation state.^[^ ^33^ ^]^ Granzyme B PET imaging can serve as a predictive biomarker for cancer immunotherapy response.^[^ ^34^, ^35^, ^36^ ^]^ Therefore, granzyme B PET imaging probe (^68^Ga‐NOTA‐GZP) was synthesized in our group based on the specific binding of granzyme B according to ref. [34] (Figure S1, Supporting Information), which can be used to assess granzyme B level real‐time in vivo.

In this study, the efficacy of KN046@^19^F‐ZIF‐8 was assessed by in vivo ^68^Ga‐NOTA‐GZP and ^18^F‐FDG micro PET/CT. Continuous uptake of ^18^F‐FDG at the tumor site was observed from days 1 to 10 after injection; only the SUV_max_ values dropped slightly from days 4 to 7 (Figure 4b). Using a ^68^Ga‐NOTA‐GZP PET/CT probe targeting granzyme B, the highest tumor tissue uptake of ^68^Ga‐NOTA‐GZP was observed 4 days after injection of the antibody drug with a tumor‐to‐normal tissue ratio (TNR) of 12 (Figure 4c), indicating the highest expression level of granzyme B after treatment by KN046@^19^F‐ZIF‐8 at day 4. Afterward, the uptake gradually decreased in the KN046 group. In contrast, the uptake of ^68^Ga‐NOTA‐GZP was sustained at day 10 in the KN046@^19^F‐ZIF‐8 group (Figure 4c). Moreover, there were significant differences in TNR based on ^68^Ga‐NOTA‐GZP PET/CT from days 4 to 10 in the KN046@^19^F‐ZIF‐8 group compared with the other three groups (*p* < 0.05) (Figure S8b, Supporting Information). In order to further confirm the granzyme B level of KN046@^19^F‐ZIF‐8 group at different time points, the granzyme B level in tumor also was measured by immunofluorescence images (Figure 4d). The granzyme B immunofluorescence staining results at different time points after KN046@^19^F‐ZIF‐8 treatment were completely consistent with the PET/CT imaging (Figure 4d). Meanwhile, the tumors of different treatment groups with the highest TNR at day 4 in ^68^Ga‐NOTA‐GZP PET/CT imaging also had the same tendency for granzyme B fluorescent staining (Figure S8a, Supporting Information). These findings indicate that ^68^Ga‐NOTA‐GZP PET accurately reflects the expression of granzyme B in tumors. The uptake of ^68^Ga‐NOTA‐GZP is positively correlated with immune activation in vivo. Therefore, granzyme B‐targeted ^68^Ga‐NOTA‐GZP PET/CT is the best non‐invasive imaging modality for real‐time monitoring of immune activation status in vivo. Moreover, KN046@^19^F‐ZIF‐8 treatment prolongs and enhances the CTL immune response, thereby significantly improving the antitumor effect of the KN046 antibody.

#### 2.5.2 In Vivo Immune Response

**Figure 5:**
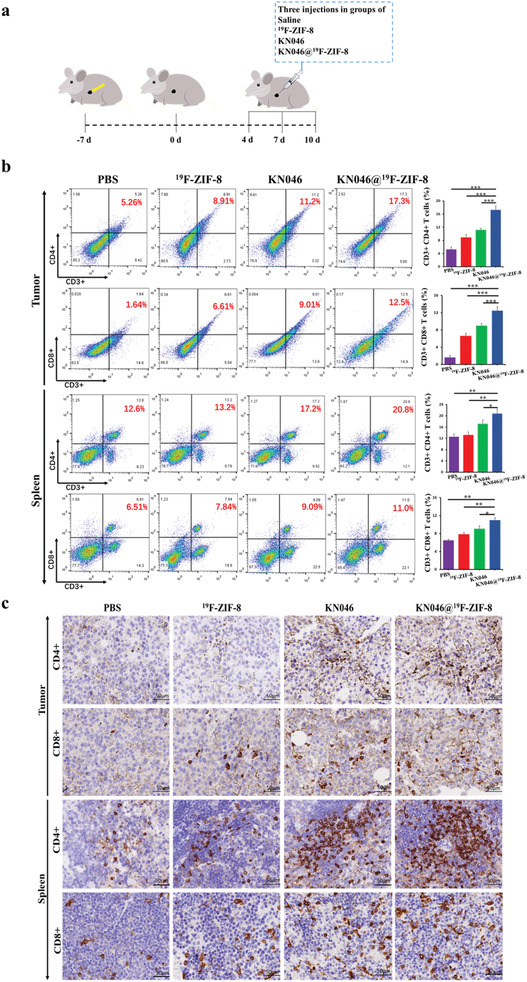
a) Schematic diagram of the therapeutic model in BALB/c mice bearing subcutaneous B16F10 melanoma tumors. b) Amounts of CD4^+^ and CD8^+^ T cells detected by flow cytometry in tumor and spleen after different treatments. Data are presented as mean ± SD (*n* = 5). *p < 0.05, **p < 0.01,***p < 0.001. c) IHC staining was used to examine CD4^+^ and CD8^+^ T cells in tumor sections and spleens at the end of the treatments (pictured at 200×) (*n* = 5).

Mice in each group were euthanized 10 days after three intratumoral injections (**Figure** **5**a). The percentages of CD4^+^ and CD8^+^ T cells in the tumor and spleen tissues were analyzed by flow cytometry. The results showed that the percentages of CD3^+^CD4^+^ T cells and CD3^+^CD8^+^ T cells in the tumor and spleen were significantly higher in the KN046@^19^F‐ZIF‐8 group than in the other groups (Figure 5b). CD4^+^ T cells are important immune regulatory cells.^[^ ^37^, ^38^ ^]^ The percentage of CD3^+^CD4^+^ cells in the spleen increased to 20.8% in the KN046@^19^F‐ZIF‐8 group compared with the normal saline (12.6%), ^19^F‐ZIF‐8 (13.2%), and KN046 groups (17.2%). The percentage of CD3^+^CD4^+^ cells in the tumor increased more than twofold in the KN046@^19^F‐ZIF‐8 group (17.3%) compared with the normal saline group (5.26%). CD8^+^ T cells are CTLs that specifically kill tumor cells.^[^ ^39^, ^40^ ^]^ The highest percentages of CD3^+^CD4^+^ and CD3^+^CD8^+^ were observed in both the spleen and tumor in the KN046@^19^F‐ZIF‐8 group, indicating that the highest efficacy among all groups was achieved in this group. The flow cytometry results were confirmed by IHC staining. As shown in Figure 5c, the numbers of CD3^+^CD4^+^ T cells and CD3^+^CD8^+^ T cells in the tumor and spleen were significantly higher in the KN046@^19^F‐ZIF‐8 group than in the other three groups.

**Figure 6:**
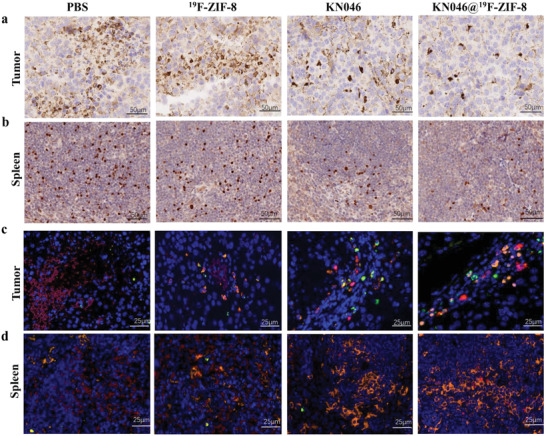
Immunohistochemical staining was used to examine Foxp3^+^ T cells in a) tumor sections and b) spleens at the end of the treatments (pictured at 200×) (*n* = 5). Multicolor immunofluorescence micrographs show the presence of CD8^+^T cells (orange), granzyme B (green), and Foxp3^+^ T cells (red) in c) tumor sections and d) spleens at the end of the treatments (pictured at 400×) (*n* = 5). DAPI (blue) indicates nuclei.

**Figure 7:**
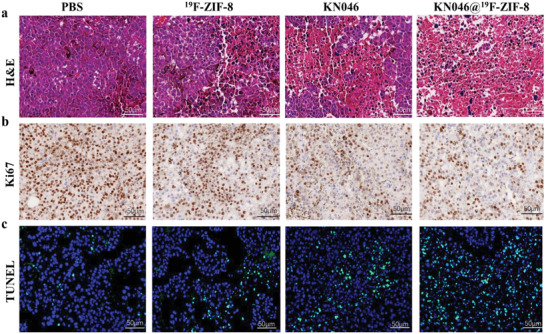
Representative micrographs of a) H&E staining, b) Ki‐67 IHC staining, and c) TUNEL immunofluorescence in tumors of each group. Cell nuclei were stained with DAPI (pictured at 200×) (*n* = 5).

In contrast, numbers of CD4^+^ Foxp3^+^ T cells—that is, regulatory T cells (Tregs) with immunosuppressive function^[^ ^41^, ^42^ ^]^—were significantly reduced in both tumor‐infiltrating lymphocytes and splenic lymphocytes in the KN046@^19^F‐ZIF‐8 group. As shown in **Figure** **6**a,b, the number of brown‐colored Foxp3^+^ T cells was significantly lower in the KN046@^19^F‐ZIF‐8 group than in the other three groups. Multicolor immunofluorescence revealed the same trends for CD8^+^ T cells, granzyme B, and Foxp3^+^ T cells in the tumor and spleen as those observed with ^68^Ga‐NOTA‐GZP PET/CT imaging, flow cytometry, and IHC staining (Figure 6c,d). Moreover, hematoxylin and eosin (H&E) (**Figure** **7**a), Ki67 IHC staining (Figure 7b), and TdT‐mediated dUTP Nick‐End Labeling (TUNEL) apoptosis staining (Figure 7c) of residual tumor tissues further confirmed the efficacy of KN046@^19^F‐ZIF‐8. Although H&E staining showed large areas of tumor necrosis in both the KN046 and KN046@^19^F‐ZIF‐8 groups, the highest percentage of necrosis was found in the KN046@^19^F‐ZIF‐8 group. As revealed by staining of Ki67, a tumor cell proliferation marker,^[^ ^43^ ^]^ KN046@^19^F‐ZIF‐8 significantly inhibited tumor tissue proliferation and differentiation. TUNEL apoptosis staining showed that the apoptosis‐positive rate was significantly higher in the KN046@^19^F‐ZIF‐8 group than the other three groups.

**Figure 8:**
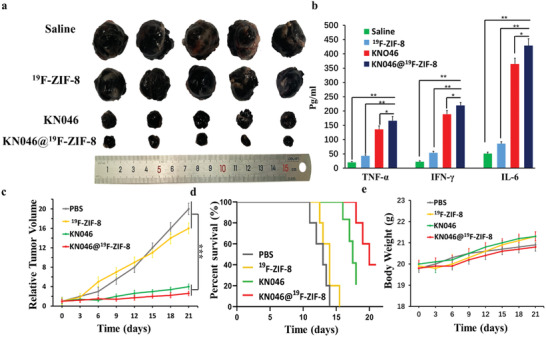
a) Photographs of resected tumors from each group 14 days after various treatments (*n* = 5). b) TNF‐*α*, IFN‐*γ*, and IL‐6 levels in the serum of mice at the end of different treatments, detected by ELISA. Data are presented as mean ± SD (*n* = 5). c) Relative tumor volume growth curves, d) survival rate, and e) weight changes in B16F10 tumor‐bearing mice during the antitumor efficacy experiments. Values are presented as mean ± SD (*n* = 5). *p < 0.05, **p < 0.01, ***p < 0.001.

Last, residual tumor photographs obtained at the end of the experiments also provided strong evidence (**Figure** **8**a). Normal saline and ^19^F‐ZIF‐8 failed to inhibit the growth of melanoma, whereas KN046 displayed slower growth, however the mice treated with KN046@^19^F‐ZIF‐8 had the smallest relative tumor volumes and the highest survival rate (Figure 8c,d). Moreover, serum TNF‐*α*, IL‐6, and IFN‐*γ* levels measured by ELISA were also significantly higher in the KN046@^19^F‐ZIF‐8 group compared with the other three groups (*p* < 0.05) (Figure 8b). All these results indicate that treatment with KN046@^19^F‐ZIF‐8 is more effective than KN046 alone. More importantly, KN046@^19^F‐ZIF‐8 provides long‐lasting PD‐L1/CTLA‐4 dual‐blockade immunotherapy to effectively induce a significantly enhanced antitumor immune response and reduce the immunosuppressive effect of tumor cells, thereby achieving higher antitumor efficacy.

The body weights of mice changed only slightly throughout the course of treatment (Figure 8e), indicating the low systemic toxicity of the treatment. The KN046@^19^F‐ZIF‐8 nanoprobe caused hemolysis of less than 2% in peripheral blood, indicating its limited in vitro toxicity (Figure S9a, Supporting Information). Compared with the normal saline group, no obvious abnormalities were seen in routine blood tests, liver and kidney function, and major organ sections in the other three treatment groups, indicating the in vivo safety of the treatment (Figure S9b–d, Supporting Information).

## 3 Conclusions

This study presents a diagnostic and therapeutic smart agent for tumor microenvironment‐responsive ^19^F MRI and dual‐blockade immunotherapy antibody delivery. The ^19^F‐doped ZIF‐8 is lysed in the tumor microenvironment to turn on the diagnostic ^19^F‐MRI nanoprobe. KN046 is a dual‐targeting inhibitor of immune checkpoints PD‐L1 and CTLA‐4 that attacks cancer cells simultaneously and powerfully from different directions. Moreover, a nano‐delivery system is used to ensure the greatest efficacy, the lowest toxicity, and the highest synergistic activity of the immune antibodies. In vivo real‐time monitoring of efficacy and controlled antibody release by ^68^Ga‐NOTA‐GZP PET/CT provides superior imaging support for exploring the mechanism of KN046 immunotherapy. Intratumoral injection of the smart nano‐agent was shown to induce systemic antitumor immunity. The combination of ^19^F‐ZIF‐8 and KN046 not only had a synergistic effect, improving pharmacokinetic efficiency and antitumor efficacy but may also delay or prevent resistance to mono‐antibody therapy. This study provides an engineering strategy for the clinical application of a more effective immunotherapy.

## 4 Experimental Section

### Reagents and Instrumentation

Unless otherwise specified, all chemicals were of analytical grade that could be used directly without further purification. KN046 was purchased from Alphamab Oncology (Suzhou, Jiangsu, China); PVP (99%), ammonium hydroxide (18%), rhodamine B, 2‐methylimidazole (99%), and GSH were from Sigma‐Aldrich (St. Louis, MO, USA); 4‐(trifluoromethyl)‐1H‐imidazole (97%) and Zn(NO_3_)·6H_2_O (99%) was from Alfa Aesar (Shanghai, China); Hoechst 33258 staining solution was from Yeasen (Shanghai, China); the CCK‐8 kit was from Dojindo Laboratories (Kumamoto, Japan); antibodies for flow cytometry were from Biolegend (California, USA); and the TUNEL apoptosis detection kit (green fluorescence) and ELISA kit were from R&D Systems (Minneapolis, Minnesota, USA). The deionized water used in all experiments was prepared using a Milli‐Q system (Millipore, Boston, MA, USA). The morphology and structure of nanoparticles were observed using a Zeiss Libra 200 SEM (Carl Zeiss, Oberkochen, Germany) and a JEM‐1230 TEM (JEOL, Tokyo, Japan). Zeta potential and DLS particle size were measured using a Nano ZS90 particle‐size analyzer (Malvern instruments, Malvern, Worcestershire, UK). UV–visible light (UV–vis) absorption spectra were recorded with a U‐3900 spectrophotometer (Hitachi, Tokyo, Japan). Fluorescence imaging was performed with a Zeiss LSM 800 CLSM (Carl Zeiss, Oberkochen, Germany). ^68^Ga‐NOTA‐GZP and ^18^F‐FDG micro PET/CT were performed using an Inveon animal PET/CT scanner (Siemens Preclinical Solution, Knoxville, TX, USA). MRI images were acquired on a 7.0 T BioSpec70/20 USR MRI system (Bruker, Karlsruhe, Germany).

### Synthesis of KN046@^19^F‐ZIF‐8

KN046 was first modified with PVP to improve its EE in nanoparticles. After the organic ligand 2‐methylimidazole in ZIF‐8 had been partially replaced by 4‐(trifluoromethyl)‐1H‐imidazole, KN046‐PVP was encapsulated in the ^19^F‐ZIF‐8 nanoshell. Briefly, PVP and KN046 were dissolved and mixed in 10 mL of deionized water at a ratio of 200:0.6 mg. Subsequently, 150 mg of 2‐methylimidazole and 50 mg of 4‐(trifluoromethyl)‐1H‐imidazole were gradually added at room temperature at 600 rpm, followed by dropwise addition of 50 µL of ammonium hydroxide. Next, 5 mL of deionized water containing 30 mg of Zn(NO_3_)_2_∙6H_2_O was slowly added to the mixture, followed by ultrasonic dispersion. The final mixture was reacted at a room temperature of 25 °C for 6 h. Finally, KN046@^19^F‐ZIF‐8 nanoparticles with a particle size of ≈100 nm were obtained after centrifugation at 8000 rpm for 10 min and washing with deionized water three times.

### Synthesis of KN046@^19^F‐ZIF‐8—Drug Loading

The modified KN046 solutions (100 µL) at different concentrations were added to 10 mL of the mixture solution of ^19^F‐ZIF‐8 under stirring. Every 6 h, 10 µL of supernatant was withdrawn for analysis of KN046 concentration at 280 nm by reversed‐phase HPLC (Infinity Agilent Technologies 1260, CA, USA). The drug loading efficiency (DL) and EE of the nanoparticles were calculated using the following formulas

(1) $$\mathrm{DL}\;\left(\%\right)=\frac{m_{\mathrm{KN}046}-V_{\mathrm{supernatant}}\times C_{\mathrm{supernatant}}}{m_{\mathrm{KN}046{@}^{19}\text{F-ZIF-8}}}\times 100\%$$

(2) $$\mathrm{EE}\;\left(\%\right)=\frac{m_{\mathrm{KN}046}-V_{\mathrm{supernatant}}\times C_{\mathrm{supernatant}}}{m_{\mathrm{KN}046}}\times 100\%$$

where *m*_KN046_ and $m_{{\text{KN046@}}^{19}\text{F-ZIF-8}}$ represented the masses of KN046 and KN046@^19^F‐ZIF‐8, respectively; and *V*_supernatant_ and *C*_supernatant_ indicated the volume and the concentration of the residual KN046 in the supernatant, respectively.

For the in vitro release study of KN046, 2 mL of KN046@^19^F‐ZIF‐8 was transferred into a dialysis bag. The dialysis bag was immersed in 30 mL of phosphate‐buffered saline (PBS) at pH 7.4, 6.5, 6.0, and 5.5, respectively, and stirred at 500 rpm in a water bath at a constant temperature of 37 °C. At selected time points, 1 mL of the external solution was withdrawn and the same volume of fresh PBS was added, followed by centrifugation at 8000 rpm for 10 min. The concentration of KN046 in the supernatant was determined by HPLC. The chromatographic conditions were as follows: buffer A (0.1% trifluoroacetic acid in acetonitrile) and buffer B (0.1% trifluoroacetic acid in water) were eluted with a gradient of 20.0–90.0% for 15.0 min; the flow rate was 1.0 mL min^−1^; and the analytical column was an Agilent PLRP‐S (5 µm, 250 × 4.6 mm).

### Cell Line and Animals

The murine B16F10 melanoma cell line was purchased from the Cell Resource Center of Shanghai Institutes for Biological Sciences, Chinese Academy of Sciences (Shanghai, China). The cells were incubated with Roswell Park Memorial Institute (RPMI) 1640 medium containing 10% fetal bovine serum and 1% penicillin/streptomycin in a 37 °C cell incubator containing 5% CO_2_. Specific‐pathogen‐free BALB/c mice (6–8 weeks old, 18–20 g) were purchased from Lingchang Biotech Co., Ltd (Shanghai, China). All in vivo experiments were carried out in accordance with the requirements of the Animal Research Committee of Fudan University on the care and use of experimental animals in research (FUSCC‐IACUC‐S20210374).

### In Vitro Cell Assay

The cytotoxicity of different concentrations (0, 25, 50, 100, 200, 400, and 800 µg mL^−1^) of KN046@^19^F‐ZIF‐8 in B16F10 cells was evaluated using a CCK‐8 assay kit according to the manufacturers’ suggested procedures. Briefly, B16F10 cells were seeded into 96‐well culture plates at a density of ≈4 × 10^4^ per well and incubated at 37 °C for 12 h. Subsequently, the cells were cultured at 37 °C for a further 48 h after adding different concentrations (0–800 µg mL^−1^) of the nanoprobe solution to each well. Afterward, 1:10 volume of CCK‐8 reagent was directly added to the cell culture medium, followed by incubation for 1–4 h. Finally, the absorbance at 450 nm was measured using a microplate reader. The experiment was repeated five times for each group.

### In Vitro Cell Assay—Cell Uptake

The distribution of KN046@^19^F‐ZIF‐8 in B16F10 cells was evaluated by CLSM. B16F10 cells were seeded on glass slides in confocal dishes at a density of 2 × 10^5^. After culture for 24 h, the medium was replaced with RPMI 1640 medium containing rhodamine‐B‐encapsulated ^19^F‐ZIF‐8. After incubation for 2–4 h in an incubator, the supernatant was removed, and the cells were washed with PBS three times before being immediately fixed in 4% paraformaldehyde, stained with Hoechst (for staining of nuclei), and imaged by CLSM.

### ^19^F MR Signal Response of KN046@^19^F‐ZIF‐8 In Vivo and In Vitro

First, a ^19^F MR signal in response to the tumor microenvironment (GSH and pH) was simulated in vitro. Briefly, PBS with different pH values (5.5, 6.0, and 7.4) was prepared. Next, 800 µL of the prepared nanoprobe solution was mixed with PBS of different pH values (5.5, 6.0, and 7.4) and GSH solutions of different concentrations (0.0, 1.0, 5.0, and 10.0 mm) in a 1.5 mL centrifuge tube. Sodium dihydrogen phosphate or disodium hydrogen phosphate was added to bring the volume to 1.0 mL to reach the target pH. The resulting mixed solutions were incubated at 37 °C for 20 min and then subjected to ^19^F MR peak measurements and ^1^H MR/^19^F MR imaging. The FLASH sequence was applied for ^19^F MRI. The RARE (rapid acquisition with refocused echoes) sequence was employed for ^1^H MRI. The parameters were as follows: repetition time (TR)/effective time (TE) = 800 ms/6.5 ms, field of view (FOV) = 7 cm × 5 cm, slice thickness = 15 mm, matrix size = 256 × 256, scan time = 1 min 16 s. The ^19^F MRI parameters were set as follows: TR/TE = 100 ms/2.26 ms, flip angle = 30°, FOV = 5 × 5 cm^2^, slice thickness = 15 mm, image size = 64× 64, averages = 100, scan time = 10 min 40 s.

### ^19^F MR Signal Response of KN046@^19^F‐ZIF‐8 In Vivo and In Vitro—^19^F MR Signal Response of Tumor Cells

B16F10 cells were seeded on confocal dishes and cultured at 37 °C overnight. After incubation with different concentrations of KN046@^19^F‐ZIF‐8 (0, 100, 200, 300, and 400 µg mL^−1^) for 6 h, the cells were washed three times with PBS to remove free nanoparticles remaining in the medium. Afterward, the cells were lysed with 300 µL of radioimmunoprecipitation assay lysis buffer for 30 min. The supernatant was then collected for ^19^F MR imaging. The imaging parameters were the same as above.

### ^19^F MR Signal Response of KN046@^19^F‐ZIF‐8 In Vivo and In Vitro—^19^F MR Signal Response in Mice

After the mice had been anesthetized with isoflurane, 150 µL of the KN046@^19^F‐ZIF‐8 solution (20 mg kg^−1^) was injected into the tumors. ^1^H/^19^F MR imaging was performed with a ^1^H/^19^F dual resonator 72‐mm volume coil at 0.5 and 2 h, respectively. The ^19^F‐FLASH imaging sequence parameters were as follows: TR/TE = 200 ms/1 ms, flip angle = 14.9°, FOV = 4 × 4 cm^2^, slice thickness = 5 mm, matrix = 32 × 32, averages = 50, scan time = 5 min 20 s.

### ^68^Ga‐NOTA‐GZP and ^18^F‐FDG Micro PET/CT for Monitoring Tumor Changes after Different Treatments

This study included two series of experiments for the assessment of antitumor efficacy. The first series was to determine the time distribution of the immune response caused by KN046@^19^F‐ZIF‐8. 7 days after subcutaneous injection of B16F10 cells on the right side at a density of 1 × 10^6^, mice (*n* = 5 in each group) were randomized into four groups to receive intratumoral injections of 100 µL of normal saline, aqueous solution containing 1 mg ZIF‐8, aqueous solution containing 1 mg KN046, or KN046@^19^F‐ZIF‐8 solution containing 1 mg KN046. ^68^Ga‐NOTA‐GZP and ^18^F‐FDG micro PET/CT were performed 0, 1, 4, 7, and 10 days after treatment to calculate the metabolic tumor volume and TNR ratios at each time point.

### ^18^F‐FDG Micro PET/CT for Monitoring Tumor Changes after Different Treatments

Tumor‐bearing mice were fasted for 6 h at predetermined time points, then injected via the tail vein with 200 µCi of ^18^F‐FDG, kept warm, anesthetized with low‐concentration isoflurane for 50 min, and imaged with micro PET/CT.

### ^68^Ga‐NOTA‐GZP Micro PET/CT for Monitoring Tumor Changes after Different Treatments

50 min after tumor‐bearing mice were injected via the tail vein with 200 µCi of ^68^Ga‐GZP at predetermined time points, micro PET/CT imaging was performed under isoflurane anesthesia. For each scan, a 5‐min CT acquisition was performed, followed by a 10‐min PET acquisition. The acquired images were reconstructed using the 3D ordered‐subset expectation maximization/maximum algorithm. The target volume was delineated without necrotic areas in tumors to calculate the tumor SUV_max_. TNR was calculated with the contralateral forelimb muscle as the background.

### Assessment of In Vivo Antitumor Efficacy after Different Treatments

The second series of experiments was to determine whether KN046@^19^F‐ZIF‐8 inhibited tumor growth. Mice were divided into four groups (*n* = 5 in each group) as tumor volumes increased to 250 mm^3^ after subcutaneous implantation of melanoma. Each group received one of the following intratumoral injections (100 µL per mouse): normal saline, ^19^F‐ZIF‐8 (1 mg), KN046 (1 mg), or KN046@^19^F‐ZIF‐8 containing 1 mg of KN046 (three injections; one injection every 3 days with 100 µL per injection). The primary tumor volume was calculated according to the following formula: width × length × height × *π*/6. Expression levels of CD3^+^CD4^+^ T cells and CD3^+^CD8^+^ T cells in the tumor and spleen were analyzed by flow cytometry. Briefly, residual tumor tissue and spleen tissue were removed, cut into small pieces of 1–2 mm, gently squeezed on a 200‐mesh sieve with a syringe holder, ground repeatedly, rinsed with PBS, and incubated with fluorescence‐labeled anticell surface antibody for 30 min at 4 °C in the dark. Moreover, changes in cytokines TNF‐*α*, IL‐6, and IFN‐*γ* in the serum were measured using ELISA kits according to the manufacturers’ suggested procedures. In addition, residual tumors and fresh spleen tissues were fixed in 4% paraformaldehyde, embedded in paraffin, and cut into 5 µm sections for PD‐L1, H&E, Ki‐67, CD4^+^, CD8^+^, and Foxp3 IHC staining, and granzyme B, TUNEL/DAPI, and multicolor immunofluorescence staining.

### Biocompatibility of KN046@^19^F‐ZIF‐8—Hemolysis Assay

Prepared nanoprobe solutions of different concentrations were mixed with 2% mouse red blood cell suspension at 37 °C and allowed to stand for 2 h. The absorbance of hemoglobin in the supernatant at 300–1000 nm was determined by UV spectrophotometry to calculate the rate of hemolysis in peripheral blood caused by the nanoprobe solution.

### Biocompatibility of KN046@^19^F‐ZIF‐8—In Vivo Safety Assessment

Normal mice were injected with KN046@^19^F‐ZIF‐8 (containing 1 mg of KN046) through the tail vein, and their vital signs were monitored for 14 days. Routine blood tests and liver and kidney function tests were also performed. The mice were euthanized 14 days later. The heart, liver, spleen, lung, and kidney were obtained and stained with H&E for pathological examination and observed under an optical microscope for toxicity.

### Statistical Analysis

All statistical tests were performed using the SPSS 24.0 software (IBM, USA). All data were presented as mean ± standard deviation (SD). Statistical comparisons of two samples were performed using Student's *t*‐test. Comparisons between multiple groups were performed using Student–Newman–Keuls test after one‐way analysis of variance. A linear model after logarithmic transformation of tumor volume was conducted to analyze the tumor growth. Kaplan–Meier method was performed to analyze differences in the animal survival distribution between treatments. All graphs were considered statistically significant when *p < 0.05, **p < 0.01, ***p < 0.001.

## Conflict of Interest

The authors declare no conflict of interest.

## Supporting information

Supporting Information

## Data Availability

Research data are not shared.
