# Supplementary material for: Engineering a Smart Agent for Enhanced Immunotherapy Effect by Simultaneously Blocking PD‐L1 and CTLA‐4
Source: Adv Sci (Weinh). 2021 Sep 2;8(20):2102500. doi: 10.1002/advs.202102500 (PMC8529437; doi:10.1002/advs.202102500)
Supplement: Supplementary file 1 — Supporting Information [file ADVS-8-2102500-s001.pdf]

© 2021 Wiley-VCH GmbH

ADVANCED SCIENCE
Open Access

# Supporting Information

for *Adv. Sci.,* DOI: 10.1002/advs.202102500

# Engineering a Smart Agent for Enhanced Immunotherapy Effect by Simultaneously Blocking PD-L1 and CTLA-4

*Chunjuan Jiang, Le Zhang, Xiaoping Xu, Ming Qi, Jianping Zhang, Simin He, Qiwei Tian,* and Shaoli Song**

# Supporting information

**Engineering a Smart Agent for Enhanced Immunotherapy Effect by Simultaneously Blocking PD-L1 and CTLA-4**

*Chunjuan Jiang, Le Zhang, Xiaoping Xu, Ming Qi, Jianping Zhang, Simin He, Qiwei Tian,* and Shaoli Song**

C. Jiang, Dr. L. Zhang, Dr. X. Xu, M. Qi, Dr. J. Zhang, S.He, Prof. S. Song
Department of Nuclear Medicine
Fudan University Cancer Hospital
Shanghai 201321, China
E-mail: shaoli-song@163.com

C. Jiang, Dr. L. Zhang, Dr. X. Xu, M. Qi, Dr. J. Zhang, S.He, Prof. S. Song
Center for Biomedical Imaging
Fudan University
Shanghai 200032, China
E-mail: shaoli-song@163.com

C. Jiang, Dr. L. Zhang, Dr. X. Xu, M. Qi, Dr. J. Zhang, S.He, Prof. S. Song
Shanghai Engineering Research Center of Molecular Imaging Probes
Shanghai 200032, China
E-mail: shaoli-song@163.com

Dr. L. Zhang, Prof. S. Song
Department of Research and Development
Shanghai Proton and Heavy Ion Center
Shanghai 201321, China
E-mail: shaoli-song@163.com

Prof. Q. Tian
Shanghai Key Laboratory of Molecular Imaging
Shanghai University of Medicine and Health Sciences
Shanghai 201318, China
E-mail: Tianqw@sumhs.edu.cn

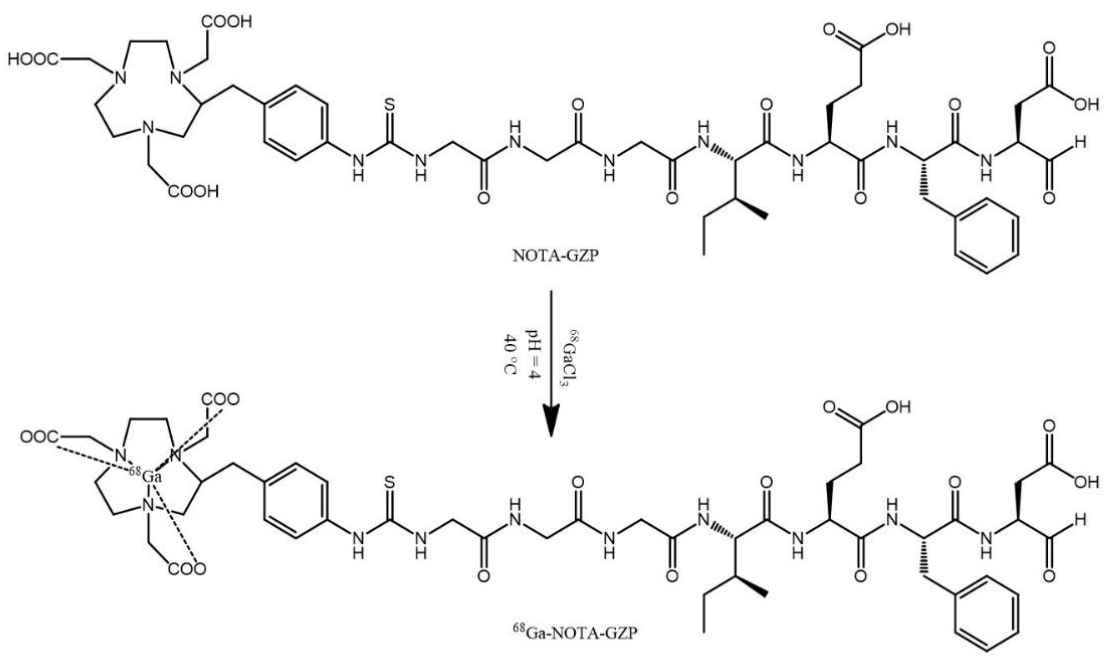


**Figure S1.** The synthetic route of $^{68}$Ga-NOTA-GZP.

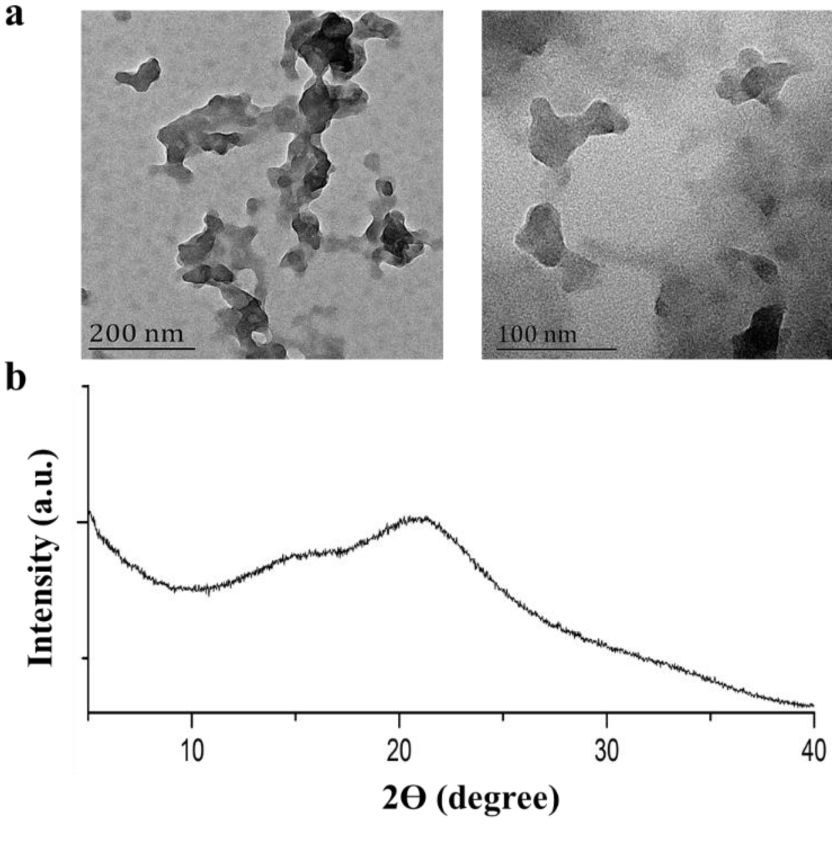


**Figure S2.** The TEM images (a) and XRD patterns (b) of KN046@$^{19}$F-ZIF-8

nanostructure dispersed in buffer solution with $C_{GSH}$=10 mM at pH=6.0.

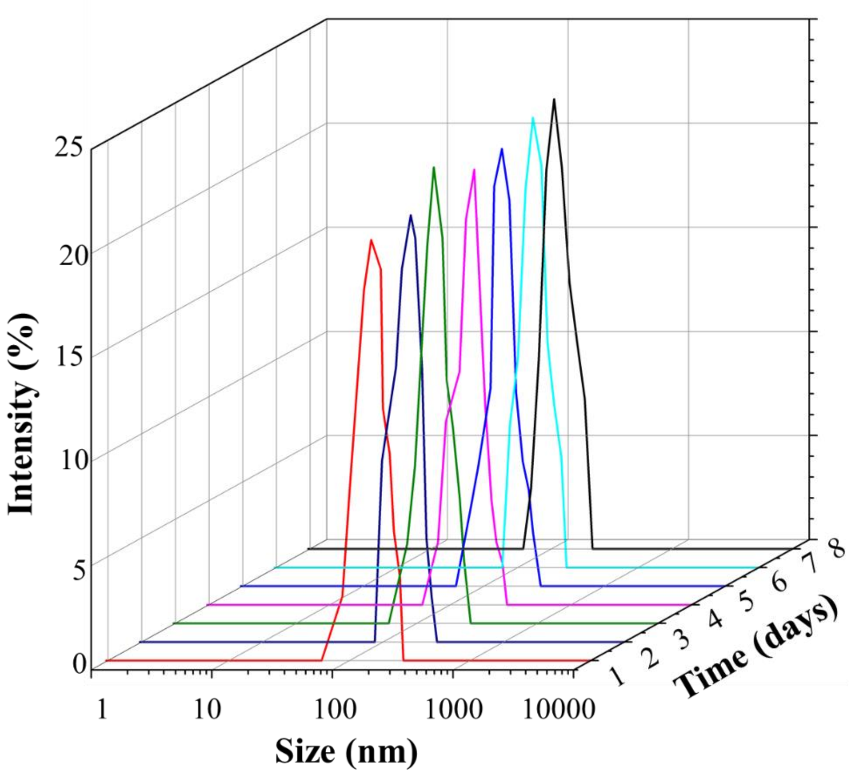


**Figure S3.** Hydrodynamic diameter of KN046@$^{19}$F-ZIF-8 in water over one week.

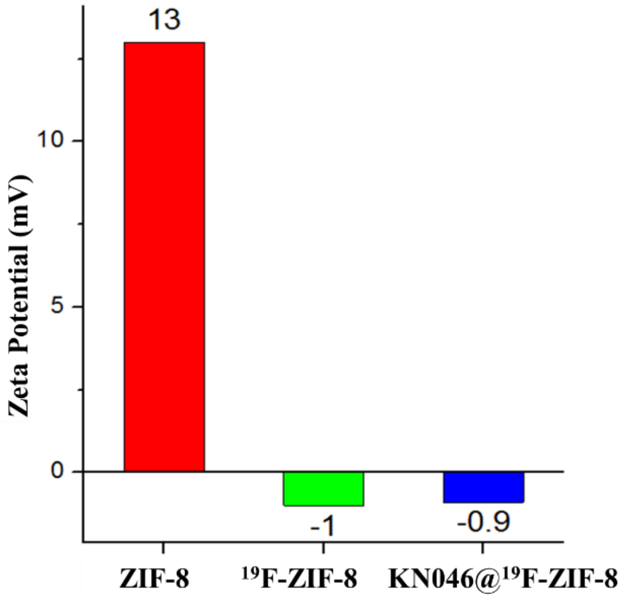


**Figure S4.** Zeta potential of ZIF-8,$^{19}$F-ZIF-8 and KN046@$^{19}$F-ZIF-8 nanoparticles.

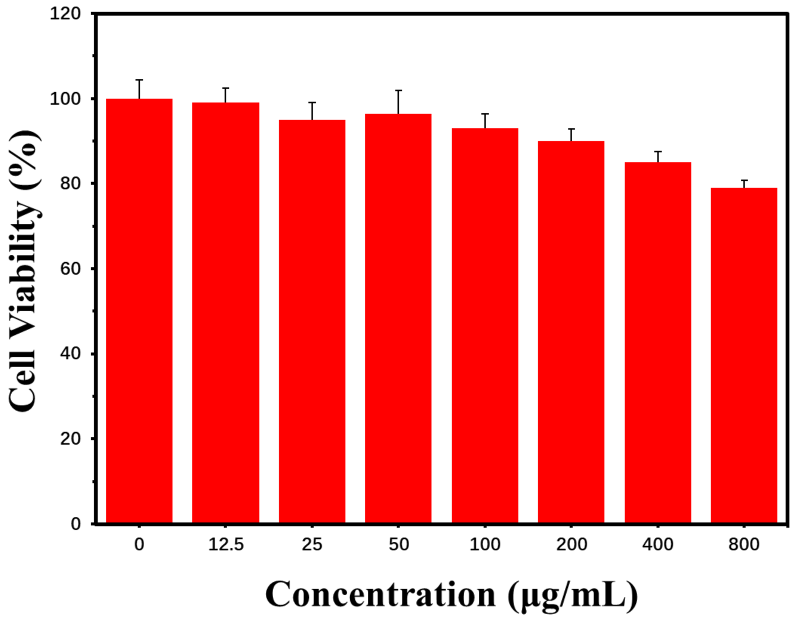


**Figure S5.** Cell viabilities of B16F10 cells after incubating with KN046@$^{19}$F-ZIF-8 at various concentrations (0–800 μg/mL) for 48 h. Quantified data were obtained from CCK-8 assays. n = 5/group.Data are presented as mean ± SD.

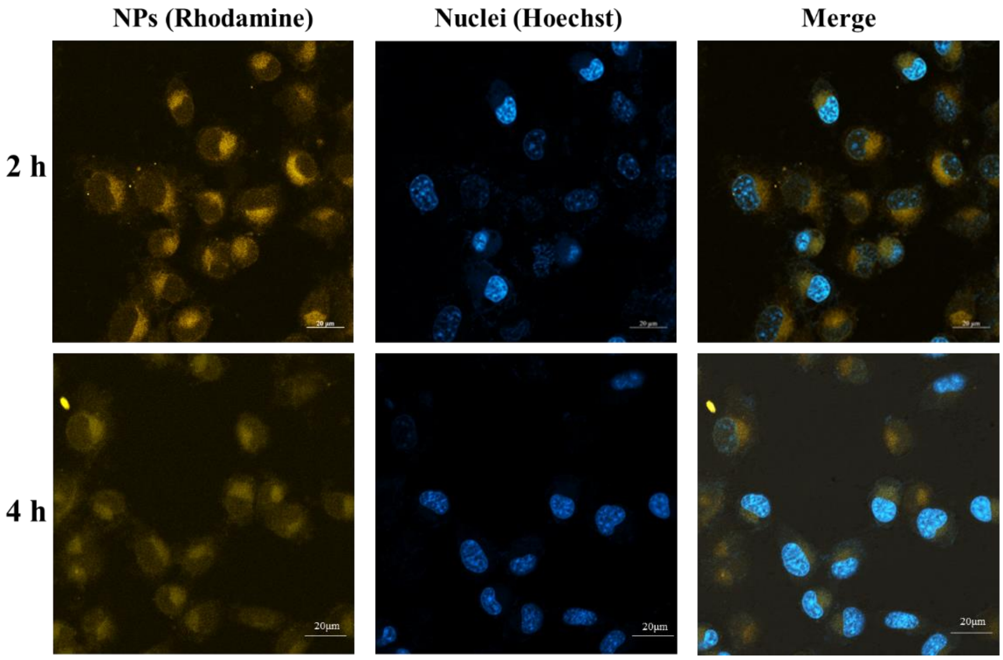


**Figure S6.** CLSM images of B16F10 cells incubated with Rh@$^{19}$F-ZIF-8 after 2 h and 4 h in the dark.Red-orange and blue signals indicate Rh@$^{19}$F-ZIF-8 and the cell

nucleus, respectively. The scale bar corresponds to 20 μm.

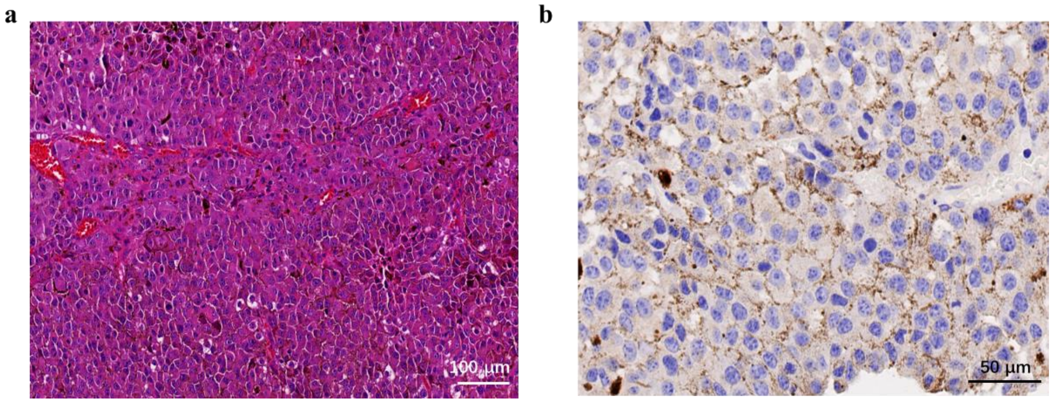


**Figure S7.** H&E staining (a) and anti-PD-L1 IHC (b) staining of B16F10 melanoma tumors.Brown staining on the cell membrane indicate the PD-L1+ cells (B).

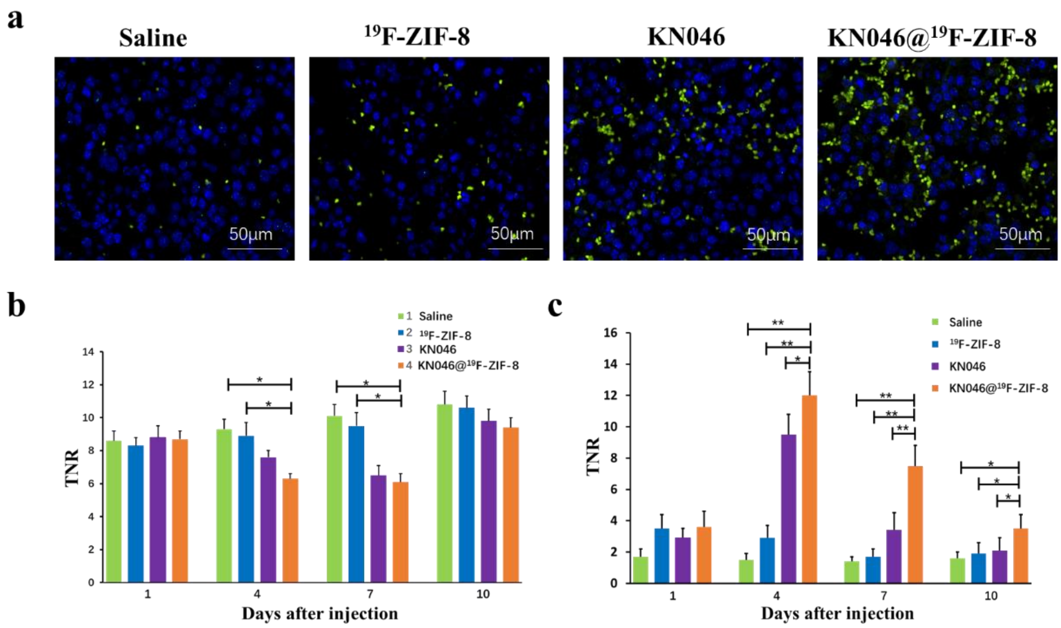


**Figure S8.** (a) Granzyme B immunofluorescence images of the tumors on day 4 after different treatments. Cell nuclei were stained with DAPI (pictured at 200 ×) (n=5).

TNRs of $^{18}$F-FDG (b) and $^{68}$Ga-NOTA-GZP (c) micro PET/CT images at different times after different treatments. Data are presented as mean ± SD (n = 5). *p < 0.05, **p < 0.01.

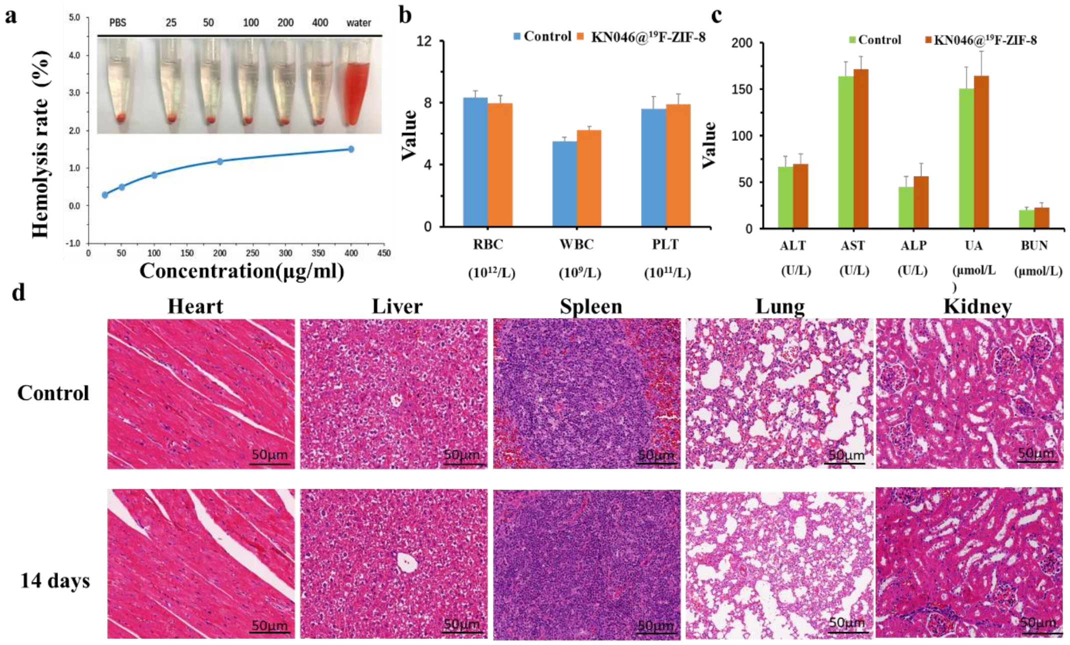


**Figure S9.** (a) Hemolysis assay of red blood cells with incubation of KN046@$^{19}$F-ZIF-8 at various concentrations for 2 h. Blood routine (b) serum biochemical index analysis (c) and H&E staining images of the major organs (d) from normal mice at 14 days after intravenous injection of KN046@$^{19}$F-ZIF-8 (100 μL,containing 1 mg of KN046) and PBS (100 μL,control),n = 5/group.
